# Electroacupuncture at 2/100 Hz Activates Antinociceptive Spinal Mechanisms Different from Those Activated by Electroacupuncture at 2 and 100 Hz in Responder Rats

**DOI:** 10.1155/2013/205316

**Published:** 2013-09-16

**Authors:** Josie Resende Torres da Silva, Marcelo Lourenço da Silva, Wiliam Alves Prado

**Affiliations:** Department of Pharmacology, Ribeirão Preto Medical School, University of São Paulo, Avenue Bandeirantes 3900, 14049-900 Ribeirão Preto, SP, Brazil

## Abstract

We examined the effects of intrathecal injection of desipramine and fluoxetine (selective inhibitors of norepinephrine and 5-HT uptake, resp.), thiorphan and neostigmine (inhibitors of enkephalinase and acetylcholinesterase, resp.), gabapentin (a GABA releaser), and vigabatrin (an inhibitor of GABA-transaminase) on the antinociception induced by 2 Hz, 100 Hz, or 2/100 Hz electroacupuncture (EA) applied bilaterally to the Zusanli (ST36) and Sanyinjiao (SP6) acupoints using the rat tail-flick test. We show that 2 Hz EA antinociception lasts longer after the administration of drugs that increase the spinal availability of norepinephrine, acetylcholine, or GABA; 100 Hz EA antinociception lasts longer after drug that increases the spinal availability of norepinephrine; 2/100 Hz EA antinociception lasts longer after drugs that increase the spinal availability of endogenous opioids or GABA. We conclude that the antinociceptive effect of 2/100 Hz EA is different from the synergistic effect of alternate stimulation at 2 and 100 Hz because the effect of the former is not changed by increasing the spinal availability of serotonin and lasts longer after the administration of vigabatrin. The combination of EA with drugs that increase the availability of spinal neurotransmitters involved in the modulation of nociceptive inputs may result in a synergistic antinociceptive effect in the rat tail-flick test.

## 1. Introduction

Electroacupuncture (EA) has been widely used to treat many different disorders and for pain relief [1]. The mechanism through which EA produces antinociception is still a matter of debate, but most authors attribute the effect to the release of endogenous opiates [2]. Stimulation of the brain structures involved with descending pain inhibitory pathways, such as the nucleus raphe magnus (NRM), nucleus gigantocellularis pars a (Gi*α*), nucleus reticularis paragigantocellularis (NRPG), locus coeruleus (LC), and subcoeruleus, potentiates EA antinociception, whereas a lesion or neural block of any of these areas attenuates EA antinociception [3]. Fibers from the aforementioned brain structures extend down to the spinal cord, passing through the dorsolateral funiculus (DLF) [4]. Supporting the notion that EA induces activation of descending pain inhibitory pathways, a DLF lesion inhibited EA antinociception in models of inflammatory [5, 6], visceral-somatic [7], and phasic [8] pain. Thus, EA antinociception may also occur by activation of noradrenergic and serotonergic descending pain inhibitory pathways [9, 10]. Serotonin (5-HT) released from NRM fibers activates spinal enkephalinergic and GABAergic neurons [11, 12], whereas spinal cholinergic [13] and GABAergic [14, 15] neurons are activated by norepinephrine (NE) released from noradrenergic fibers.

The mechanisms activated by EA may differ according to the frequency of stimulation. Low-frequency EA (<15 Hz) increases the spinal release of met-enkephalin, endomorphin and beta-endorphin, while high-frequency EA (15 to 100 Hz) increases the spinal release of dynorphin [1, 2]. Alternate stimulation at low (2 Hz) and high (100 Hz) frequencies (2/100 Hz EA) has also been proposed as a way to elicit antinociceptive effects [2]. In fact, 2/100 Hz EA was followed by an increased release of dynorphin and endomorphin in the spinal fluid, and its antinociceptive effect did not occur after intrathecal administration of selective *k*- or *μ*-opioid-receptor antagonists [1, 16]. 

We have recently utilized intrathecal antagonists of these spinal mediators to confirm that 2 and 100 Hz EA [17] and 2/100 Hz EA [18] activate different descending mechanisms in the spinal cord to induce antinociception in the rat tail-flick test. The present study further comparatively evaluates the effects of intrathecal injection of desipramine [19] and fluoxetine [20] (selective inhibitors of norepinephrine and 5-HT uptake, resp.), thiorphan [21] and neostigmine [22] (inhibitors of enkephalinase and acetylcholinesterase, resp.), gabapentin (a GABA releaser) [23, 24], and vigabatrine (an inhibitor of GABA-transaminase) [25] on the antinociception induced by 2 Hz, 100 Hz, or 2/100 Hz EA in the rat tail-flick test.

## 2. Materials and Methods

### 2.1. Subjects

Male Wistar rats (140–160 g) were used in this study. Animals were housed two per cage under controlled temperature (24 ± 2°C) and on a 12 h light-dark cycle, with the dark cycle beginning at 07:00 h. Animals had free access to food and water. The experiments were approved by the Commission of Ethics in Animal Research, Faculty of Medicine of Ribeirão Preto, University of São Paulo (number 026/2010). The guidelines of the Committee for Research and Ethical Issues of IASP [26] were followed throughout the experiments. Each rat was used only once.

### 2.2. Tail-Flick Test

Each animal was placed in a ventilated tube with the tail laid across a wire coil maintained at room temperature (22 ± 1°C). The coil temperature was then raised by the passage of electric current, and the latency for the tail-withdrawal reflex was measured. Heat was applied to a portion of the ventral surface of the tail between 4 and 6 cm from the tip. Each trial was terminated after 6 seconds to minimize the possibility of skin damage. Tail-flick latency (TFL) was measured at 5-minute intervals until a stable baseline was obtained in 3 consecutive trials. Only rats showing stable baseline latency after up to 6 trials were used in each experiment. Anesthetized rats were maintained on a wooden plate while TFL was measured.

### 2.3. Selection of Animals

Individual differences have been reported in rodents regarding the antinociceptive effect of EA [27], and thus, animals can be classified as responders or nonresponders [28]. Therefore, pharmacological treatments that reduce the antinociceptive efficacy of EA may be misinterpreted if non-responder and low-responder rats are pooled in the same experimental group. Each animal used in the experiments was therefore preliminarily submitted to the tail-flick test to determine its baseline TFL. The rat was lightly anesthetized with isoflurane in oxygen flow through a loose-fitting, cone-shaped mask, 2% for induction and 0.5% for maintenance. The tail-flick test was repeated 5 min later, and a 10 min period of EA was applied bilaterally to the Zusanli (ST36) and Sanyinjiao (SP6) acupoints at a frequency of 2 Hz, as described elsewhere [8]. We considered an increase of at least 90% in TFL (>5.5 s) as the minimum to classify positive responders to the effect of EA, as proposed elsewhere [29]. The responder rats were selected for further experiments one week later. 

### 2.4. Electroacupuncture

The procedures were performed on lightly anesthetized rats as described above to minimize the stress induced by the animal restraint that is necessary for needle insertion and stimulation [30]. Uninsulated stainless steel (type I) acupuncture needles (0.18 × 8 mm, Dong Bang Acupuncture Inc., Chungnam, Korea) were inserted bilaterally at a depth of 5 mm into each hind leg at ST36 and SP6. The stimuli were generated by a constant current pulse generator model EL-608 (NKL, Brusque, SC, Brazil) and applied for 20 min to both hind legs simultaneously. The electric stimuli were set as square waves with a 5-ms width and a frequency of 2, 100, or 2/100 Hz. The 2/100 Hz frequency consisted of the application of EA at 2 Hz alternating with 100 Hz, each lasting 3 seconds. The current intensity was increased in a stepwise manner until a muscle twitch was observed (140–150 *μ*A) [31, 32]. EA was then performed in each rat at a current intensity of 1.4–1.5 mA, which corresponds to 10 times the muscle twitch threshold [33]. Animals allocated into the sham EA groups were placed in the same apparatus and had needle insertion in the same acupoints, but no electrical current was applied to them. 

### 2.5. Intrathecal Injection

The injections were performed in lightly anesthetized rats using a 1 inch, 25-G needle that was transcutaneously introduced at the L5-L6 level into the subarachnoid space [34]. A sudden lateral movement of the tail was considered to be indicative that the needle had entered the subarachnoidal space. A constant 10 *μ*L volume was injected, and the syringe was then held in position for a few seconds and gradually removed to avoid any outflow of the drug. All solutions contained 1% fast green dye to confirm the correct position of the injection. Each rat received only one intrathecal injection.

### 2.6. Experimental Protocol

**Figure 1:**
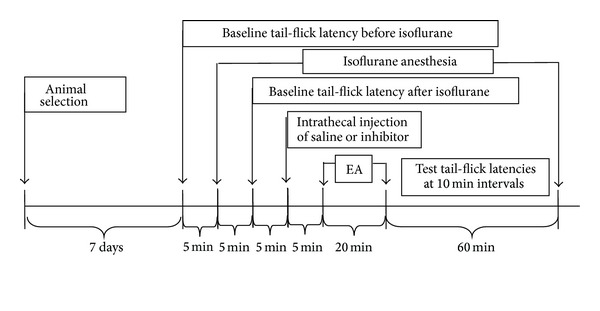
Summary of the protocol used in the study (EA = electroacupuncture).

All experiments were conducted in groups of six responder rats. The protocol used in this study is summarized in Figure 1. Rats were first selected for the determination of baseline TFL. Each animal was then anesthetized with isoflurane, and TFL was measured 5 minutes later. Only rats showing similar pre- and postanesthetic TFL were considered for further analysis. Saline or drug was then injected intrathecally, and 5 minutes later, TFL was measured once again. Rats were subsequently submitted to a 20 minute period of EA, and TFL was measured within 30 seconds after the end of stimulation and at 10 minute intervals for up to 60 minutes. No attempt was made to measure tail-flick latency during the EA period. 

### 2.7. Drugs

The following drugs were used: desipramine hydrochloride, fluoxetine hydrochloride, dl-thiorphan, and neostigmine bromide were obtained from Sigma-Aldrich (St. Louis, MO); gabapentin and vigabatrine were obtained from Tocris Bioscience (Ellisville, MO). All drugs were dissolved in saline.

### 2.8. Histology

At the end of the experiment, each animal was deeply anesthetized with intraperitoneal sodium thiopental and perfused through the heart with 4% paraformaldehyde in 0.1 M phosphate buffered saline. Incorrect injections were detected by the presence of fast green dye in the paravertebral musculature or in the dorsal subcutaneous region. Rats found to have been given an incorrect injection were not used for further data analysis.

### 2.9. Statistical Analysis

The tail-flick latencies are presented in graphs as the mean ± standard deviation (SD). Comparisons between control and test groups were made by multivariate analysis of variance (MANOVA) with repeated measures to compare the groups over all time periods.

The factors analyzed were treatment, time, and treatment *X* time interaction. In the case of a significant treatment *X* time interaction, 1 way analysis of variance followed by the Bonferroni post hoc test was performed for each time point. The post-EA times during which the TFL of drug- and saline-treated rats was significantly different were compared using ANOVA followed by Tukey's multiple comparison tests. Statistical analysis was performed using the statistical software package SPSS/PC+, version 17.0 (SPSS Inc., Chicago, IL, USA). A probability value of *P* < 0.05 was considered to be statistically significant.

## 3. Results

### 3.1. Responder Rats and the Measurement of Pain Thresholds

Four hundred and eighty rats were initially used to determine whether they developed antinociception in response to EA. Three hundred and twelve rats (65%) were classified as responders, and 168 were nonresponders (35%). Only responder rats were used in the subsequent experiments.

**Figure 2:**
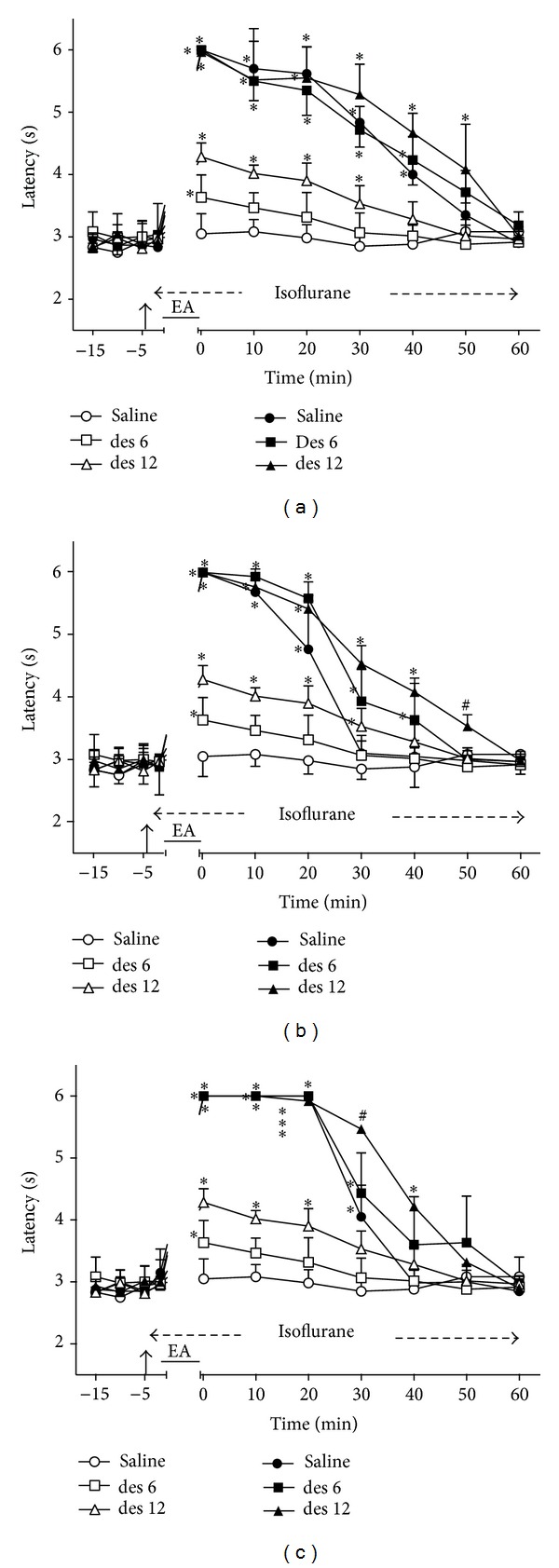
Time course of the changes induced by intrathecal desipramine (des) or saline (10 *μ*L) on antinociception induced by sham (open symbols) or real (black symbols) electroacupuncture (EA) at 2 (a), 100 (b), or 2/100 (c) Hz frequency on the tail-flick latency of rats anesthetized with isoflurane. Anesthesia was performed during the period indicated by a horizontal dashed arrow. Desipramine (6 or 12 *μ*g/10 *μ*L) was injected at the time indicated by a vertical arrow. EA was applied for 20 minutes (horizontal line). Points are means (± SD) of 6 rats per group. *P* < .05 compared to saline/sham EA (∗) using the Bonferroni post hoc test.

**Figure 3:**
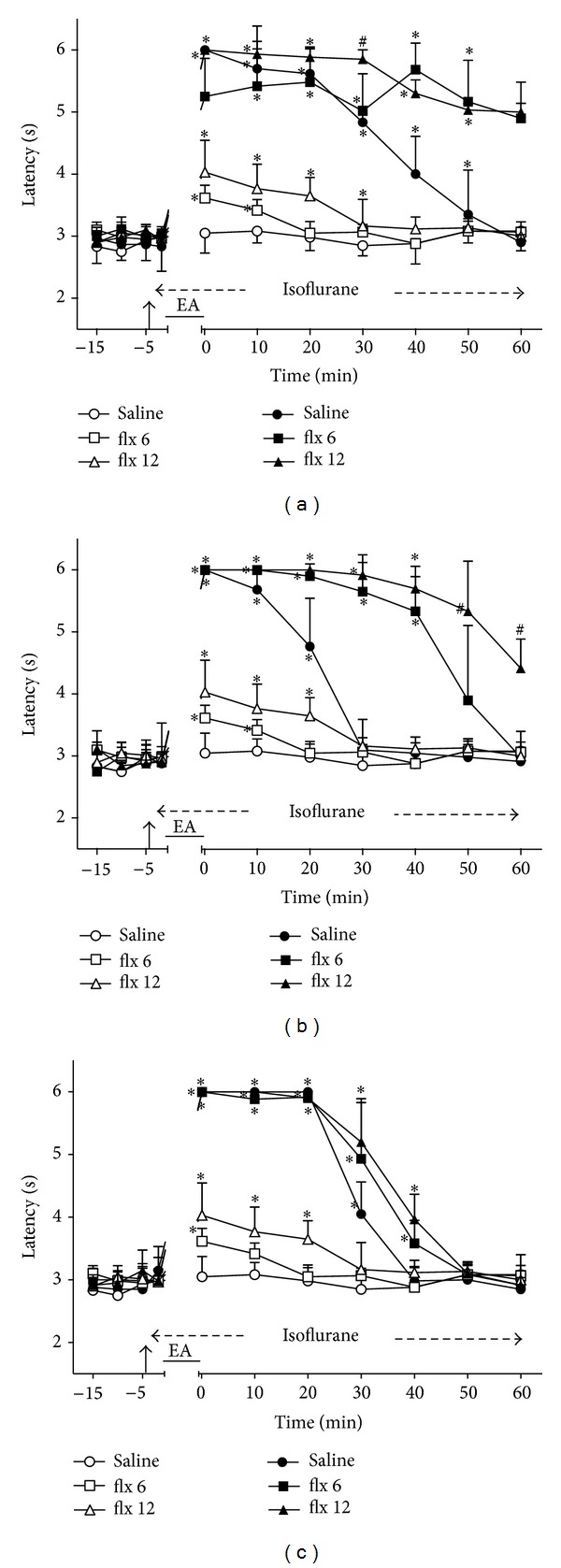
Time course of the changes induced by intrathecal fluoxetine (flx) or saline (10 *μ*L) on antinociception induced by sham (open symbols) or real (black symbols) electroacupuncture (EA) at 2 (a), 100 (b), or 2/100 (c) Hz frequency on the tail-flick latency of rats anesthetized with isoflurane. Anesthesia was performed during the period indicated by a horizontal dashed arrow. Fluoxetine (6 or 12 *μ*g/10 *μ*L) was injected at the time indicated by a vertical arrow. EA was applied for 20 minutes (horizontal line). Points are means (± SD) of 6 rats per group. *P* < .05 compared to saline/sham EA (∗) using the Bonferroni post hoc test.

**Figure 4:**
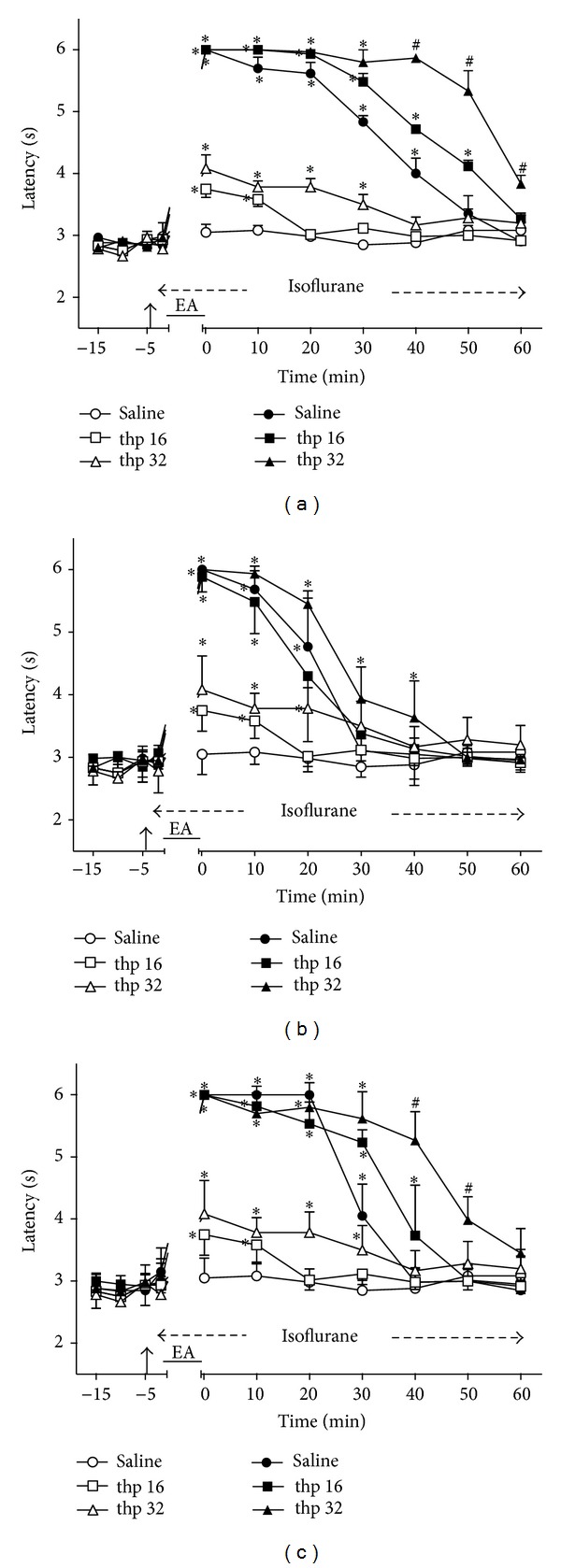
Time course of the changes induced by intrathecal thiorphan (thp) or saline (10 *μ*L) on antinociception induced by sham (open symbols) or real (black symbols) electroacupuncture (EA) at 2 (a), 100 (b), or 2/100 (c) Hz frequency on the tail-flick latency of rats anesthetized with isoflurane. Anesthesia was performed during the period indicated by a horizontal dashed arrow. Thiorphan (16 or 32 *μ*g/10 *μ*L) was injected at the time indicated by a vertical arrow. EA was applied for 20 minutes (horizontal line). Points are means (± SD) of 6 rats per group. *P* < .05 compared to saline/sham EA (∗) or any other group (#) using the Bonferroni post hoc test.

**Figure 5:**
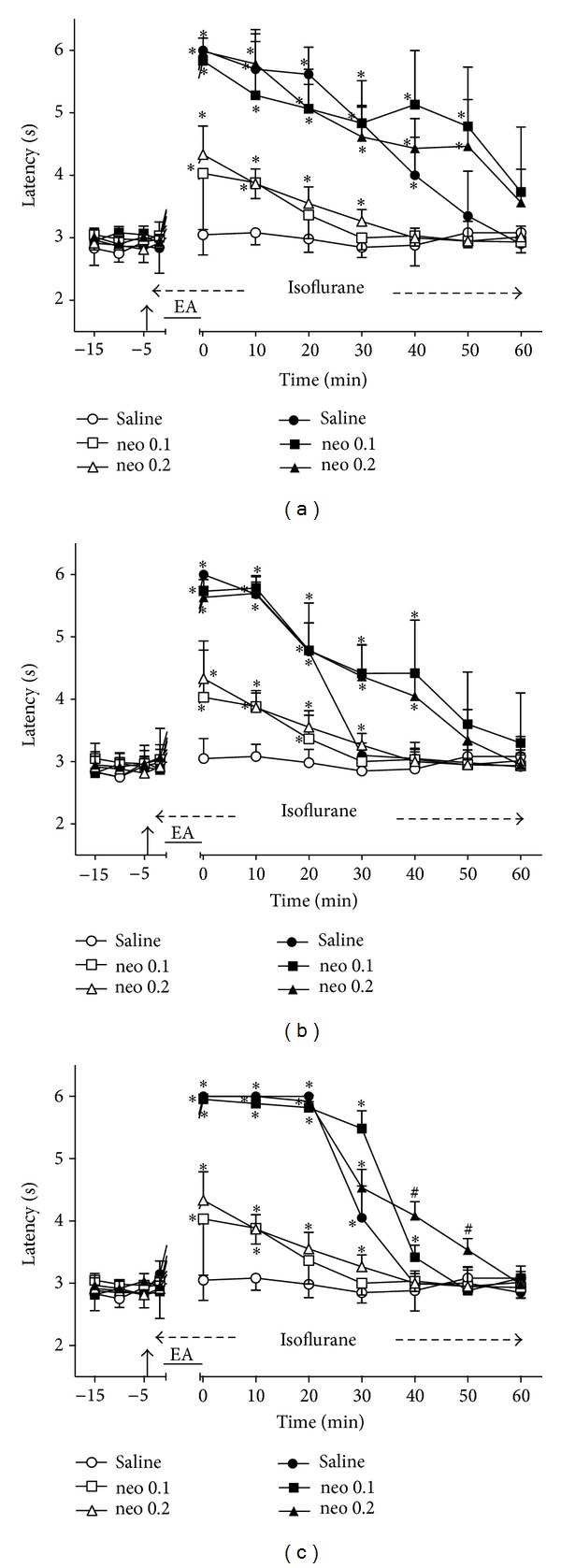
Time course of the changes induced by intrathecal neostigmine (neo) or saline (10 *μ*L) on antinociception induced by sham (open symbols) or real (black symbols) electroacupuncture (EA) at 2 (a), 100 (b), or 2/100 (c) Hz frequency on the tail-flick latency of rats anesthetized with isoflurane. Anesthesia was performed during the period indicated by a horizontal dashed arrow. Neostigmine (0.1 or 0.2 *μ*g/10 *μ*L) was injected at the time indicated by a vertical arrow. EA was applied for 20 minutes (horizontal line). Points are means (± SD) of 6 rats per group. *P* < .05 compared to saline/sham EA (∗) or any other group (#) using the Bonferroni post hoc test.

**Figure 6:**
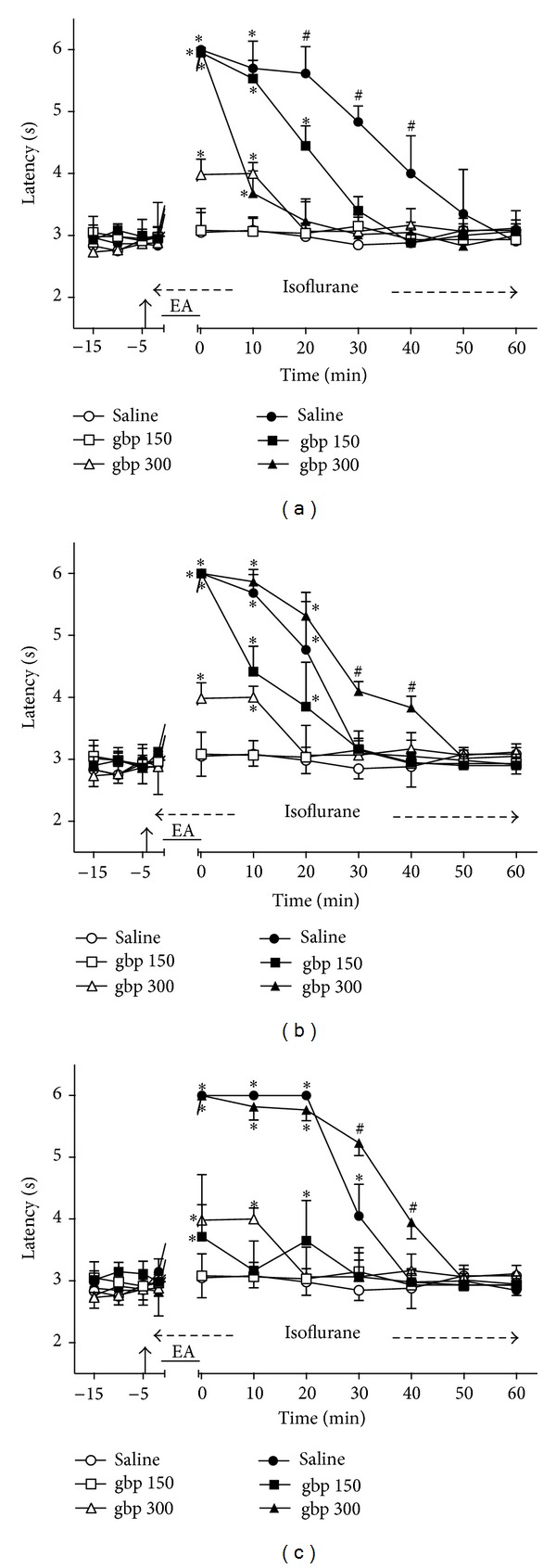
Time course of the changes induced by intrathecal gabapentin (gbp) or saline (10 *μ*L) on antinociception induced by sham (open symbols) or real (black symbols) electroacupuncture (EA) at 2 (a), 100 (b), or 2/100 (c) Hz frequency on the tail-flick latency of rats anesthetized with isoflurane. Anesthesia was performed during the period indicated by a horizontal dashed arrow. Gabapentin (150 or 300 *μ*g/10 *μ*L) was injected at the time indicated by a vertical arrow. EA was applied for 20 minutes (horizontal line). Points are means (± SD) of 6 rats per group. *P* < .05 compared to saline/sham EA (∗) or any other group (#) using the Bonferroni post hoc test.

**Figure 7:**
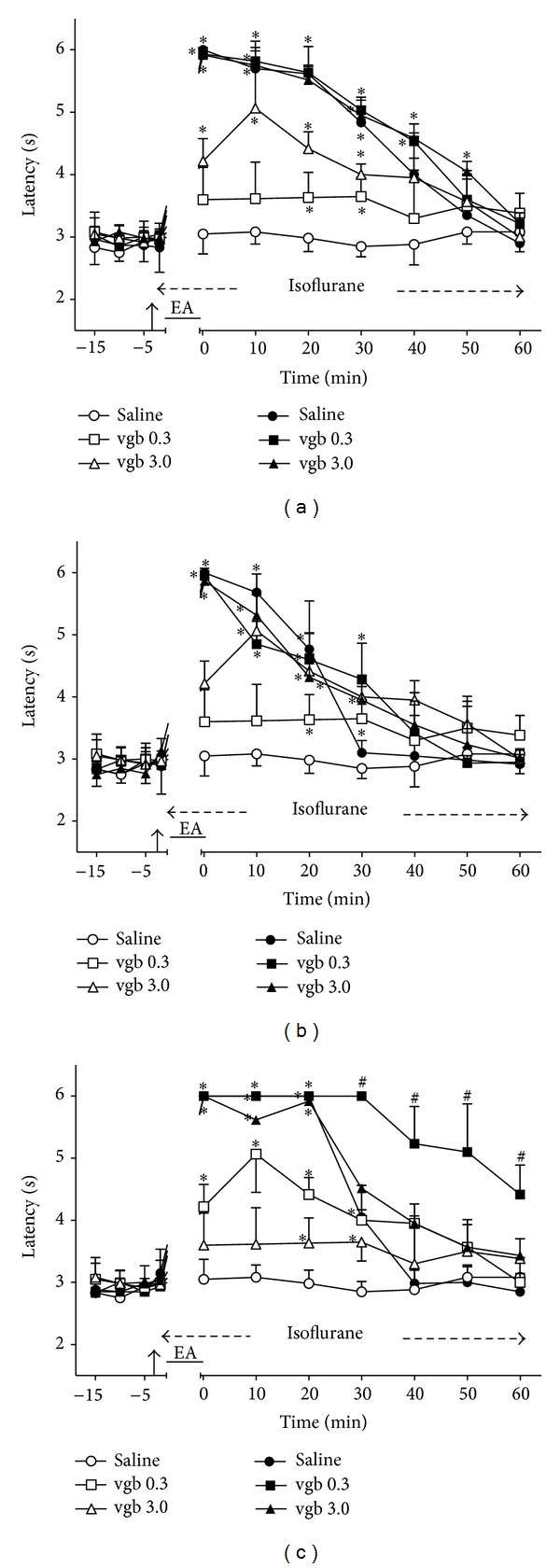
Time course of the changes induced by intrathecal vigabatrin (vgb) or saline (10 *μ*L) on antinociception induced by sham (open symbols) or real (black symbols) electroacupuncture (EA) at 2 (a), 100 (b), or 2/100 (c) Hz frequency on the tail-flick latency of rats anesthetized with isoflurane. Anesthesia was performed during the period indicated by a horizontal dashed arrow. Vigabatrine (0.3 or 3.0 *μ*g/10 *μ*L) was injected at the time indicated by a vertical arrow. EA was applied for 20 minutes (horizontal line). Points are means (± SD) of 6 rats per group. *P* < .05 compared to saline/sham EA (∗) or any other group (#) using the Bonferroni post hoc test.

The results of the experiments conducted with rats treated with desipramine, fluoxetine, thiorphan, neostigmine, gabapentin, and vigabatrine are shown in Figures 2, 3, 4, 5, 6, and 7. The experimental groups did not differ significantly in the baseline TFL measured before surgery. The latencies before and 5 min after exposure to isoflurane in each group were not significantly different among the experiments. The rats tolerated needle insertion and EA stimulation well under 0.5% isoflurane.

Sham EA did not influence TFL in saline-treated rats, and this group served as a control. EA applied to saline-treated rats at a 2, 100, or 2/100 Hz frequency induced a long-lasting inhibition of the tail-flick reflex (Part A, B, or C, resp., of Figures 2 to 7), and these groups served as controls for drug-treated rats. The maximum possible effect in the test (TFL = 6 s) was observed soon after the end of the period of stimulation at all frequencies. The maximum possible effect lasted less than 10 min after 2 Hz EA but was longer after 100 Hz EA (at least 10 min) and 2/100 Hz EA (at least 20 min). The TFL remained significantly above that of the control for at least 40 min after 2 and 100 Hz EA and for at least 30 min after 2/100 Hz EA. 

### 3.2. Time Course of the Changes Induced by Intrathecal Desipramine on EA Effects

Intrathecal desipramine (6 and 12 *μ*g) followed by sham EA produced a dose-dependent and transient increase of TFL (Figure 2). The effect was significantly different from that of the control for less than 10 min after the smaller dose and for at least 30 min after the higher dose. These two groups served as controls for the remaining experiments with desipramine (Figures 2(a)–2(c)). The maximum possible TFL was still reached when 2 (Figure 2(a)), 100 (Figure 2(b)) or 2/100 (Figure 2(c)) Hz EA was applied to desipramine-treated rats. The curves in Figures 2(a), 2(b), and 2(c) are significantly different with regard to treatment (*F*_5,30_ = 126.40, 131.61, and 229.95, resp.) and time (*F*_10,300_ = 220.41, 321.85, and 327.51, resp.) and show a significant treatment *X* time interaction (*F*_50,300_ = 22.08, 35.25, and 35.63, resp.). *P* < .001 in all cases.

### 3.3. Time Course of the Changes Induced by Intrathecal Fluoxetine on EA Effects

Intrathecal fluoxetine (6 and 12 *μ*g) followed by sham EA produced a dose-dependent and transient increase of TFL (Figure 3). The effects were significantly different from those in the control group for at least 10 min after the smaller dose and 30 min after the higher dose. These two groups served as controls for the remaining experiments with fluoxetine (Figures 3(a)–3(c)). The intensity of the effects of 2 (Figure 3(a)), 100 (Figure 3(b)), or 2/100 (Figure 3(c)) Hz EA in fluoxetine- and saline-treated rats was not significantly different. However, the duration of the antinociceptive effects of 2 Hz EA lasted for at least 60 min in rats treated with the lower or higher dose of fluoxetine (Figure 3(a)). The antinociceptive effect of 100 Hz EA lasted 40 min in rats treated with fluoxetine (6 *μ*g) and at least 60 min in rats treated with fluoxetine (12 *μ*g) (Figure 3(b)). The antinociceptive effect of 2/100 Hz EA lasted longer (40 min) in fluoxetine-treated (6 or 12 *μ*g) rats than in saline-treated rats (Figure 3(c)). The curves in Figures 3(a), 3(b), and 3(c) are significantly different with regard to treatment (*F*_5,30_ = 264.47, 168.62, and 168.25, resp.) and time (*F*_10,300_ = 169.68, 208.23, and 310.11, resp.) and show a significant treatment *X* time interaction (*F*_50,300_ = 27.30, 35.93, and 40.53, resp.). *P* < .001 in all cases.

### 3.4. Time Course of the Changes Induced by Intrathecal Thiorphan on EA Effects

Intrathecal thiorphan (16 and 32 *μ*g) followed by sham EA also produced a dose-dependent and transient increase of TFL (Figure 4). The effects were significantly different from those of the control group for at least 10 min after the smaller dose and 30 min after the higher dose. These two groups served as controls for the remaining experiments with thiorphan (Figures 4(a)–4(c)). The intensity of the effects of 2 (Figure 4(a)), 100 (Figure 4(b)), or 2/100 (Figure 4(c)) Hz EA in thiorphan-treated (16 and 32 *μ*g) and saline-treated rats was not significantly different. The curves in Figures 4(a), 4(b), and 4(c) are significantly different with regard to treatment (*F*_5,30_ = 359.69, 74.96, and 79.46, resp.) and time (*F*_10,300_ = 287.39, 204.69, and 290.85, resp.) and show a significant treatment *X* time interaction (*F*_50,300_ = 34.35, 20.20, and 35.34, resp.). *P* < .001 in all cases.

### 3.5. Time Course of the Changes Induced by Intrathecal Neostigmine on EA Effects

Intrathecal neostigmine (0.1 and 0.2 *μ*g) followed by sham EA also produced a dose-dependent and transient increase of TFL (Figure 5). The effects were significantly different from those of the control group for at least 10 min after the smaller dose and 30 min after the higher dose of neostigmine. These two groups served as controls for the remaining experiments with neostigmine (Figures 5(a)–5(c)). The intensity of the antinociceptive effects of 2 (Figure 5(a)), 100 (Figure 5(b)), or 2/100 (Figure 5(c)) Hz EA in neostigmine- and in saline-treated rats did not differ significantly. The curves in Figures 5(a), 5(b), and 5(c) are significantly different with regard to treatment (*F*_5,30_ = 50.08, 60.47, and 163.83, resp.) and time (*F*_10,300_ = 130.55, 172.24, and 362.65, resp.) and show a significant treatment *X* time interaction (*F*_50,300_ = 13.39, 15.32, and 37.29, resp.). *P* < .001 in all cases.

### 3.6. Time Course of the Changes Induced by Intrathecal Gabapentin on EA Effects

Intrathecal gabapentin (300 *μ*g) followed by sham EA produced a significant but short-lasting (at least 10 min) increase of TFL (Figure 6). In contrast, sham EA in gabapentin (150 *μ*g)-treated rats did not produce significant changes in TFL. The rats treated with gabapentin (150 and 300 *μ*g) followed by sham EA were used as controls for the remaining experiments with gabapentin (Figures 6(a)–6(c)). The intensity of the antinociceptive effect of 2 (Figure 6(a)) and 100 (Figure 6(b)), Hz EA in gabapentin-treated (150 and 300 *μ*g) and saline-treated rats did not differ significantly. However, the effect of 2/100 Hz EA was blocked by the lower dose of gabapentin (Figure 6(c)). The curves in Figures 6(a), 6(b), and 6(c) are significantly different with regard to treatment (*F*_5,30_ = 117.12, 102.45, and 146.00, resp.) and time (*F*_10,300_ = 203.82, 228.12, and 159.84, resp.) and show a significant treatment *X* time interaction (*F*_50,300_ = 34.92, 32.14, and 36.95, resp.). *P* < .001 in all cases.

### 3.7. Time Course of the Changes Induced by Intrathecal Vigabatrin on EA Effects

Intrathecal vigabatrin (3.0 *μ*g) followed by sham EA produced a significant increase of TFL lasting at least 30 min (Figure 7). In contrast, sham EA in vigabatrin (0.3 *μ*g)-treated rats produced a slight increase in TFL that was significantly different from that of the control group at 20 and 30 min after EA. The rats treated with vigabatrin (0.3 and 3.0 *μ*g) followed by sham EA were then used as controls for the remaining experiments with vigabatrin (Figures 7(a)–7(c)). The intensity of the antinociceptive effect of 2 (Figure 7(a)), 100 (Figure 7(b)), and 2/100 (Figure 7(c)) Hz EA in vigabatrin-treated (0.3 and 3.0 *μ*g) rats and in saline-treated rats did not differ significantly. The curves in Figures 7(a), 7(b), and 7(c) are significantly different with regard to treatment (*F*_5,30_ = 113.16, 29.66, and 233.05, resp.) and time (*F*_10,300_ = 21.47, 157.05, and 26.35, resp.) and show a significant treatment *X* time interaction (*F*_50,300_ = 249.07, 15.19, and 123.57, resp.). *P* < .001 in all cases.

### 3.8. Duration of Effects of EA of Different Frequencies on Pain Threshold after Drug Injection

**Figure 8:**
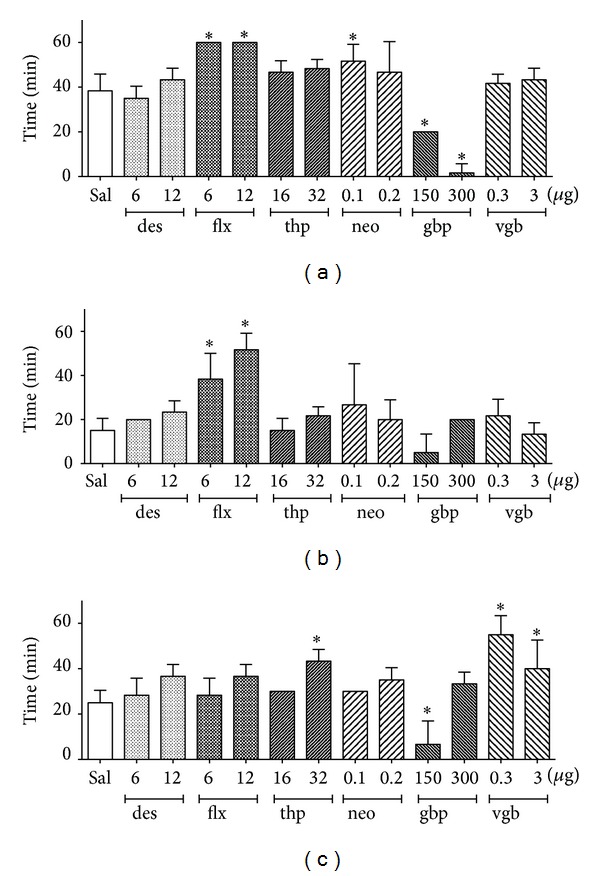
Time courses (minutes) after a 20 min period of electroacupuncture applied at 2 (a), 100 (b), or 2/100 (c), Hz frequency during which the tail-flick latencies of drug- and saline-treated rats were significantly different were compared using ANOVA followed by Tukey's multiple comparison test. Abbreviations: des = desipramine; flx = fluoxetine; thp = thiorphan; neo = neostigmine; gbp = gabapentin; vgb = vigabatrin. Doses are indicated in *μ*g/10 *μ*L. (∗) *P* < .05 compared to saline.

The post-EA times during which the TFL of saline- or drug-treated rats was significantly different from that of the EA sham groups were also compared, and the results are shown in Figures 8(a), 8(b), and 8(c) for 2, 100, and 2/100 Hz EA, respectively. The duration of the effect of 2 Hz EA was significantly longer in fluoxetine (6 and 12 *μ*g) treated and neostigmine (0.1 *μ*g) treated rats and shorter in gabapentin (150 and 300 *μ*g) treated rats compared with saline-treated rats. The duration of the effect of 100 Hz EA was significantly longer in fluoxetine (6 and 12 *μ*g) treated rats than in saline-treated rats. The duration of the effect of 2/100 Hz EA was significantly longer in thiorphan (32 *μ*g) treated and vigabatrin (0.3 and 3 *μ*g)-treated rats than in saline-treated rats. In contrast, rats treated with gabapentin (150 *μ*g) had a significantly shorter duration of 2/100 Hz EA-induced antinociception than rats treated with saline. The durations of the antinociceptive effects of 2 (Figure 8(a)), 100 (Figure 8(b)), and 2/100 (Figure 8(c)) Hz EA after the remaining treatments were not significantly different from those obtained in saline-treated rats. 

## 4. Discussion

### 4.1. The Effect of EA on Responder Rats under Inhalation Anesthesia with Isoflurane

Sixty-five percent of the rats initially screened for this study were responders to EA-induced antinociception using the tail-flick test, a proportion analogous to that described elsewhere [35, 36]. TFL was significantly increased by EA at 2, 100, or 2/100 Hz. The antinociceptive effect obtained following 2 or 100 Hz EA remained near the cut-off level for as long as 30 minutes in the majority of the tested rats. In contrast, all rats reached the cut-off level for at least 20 min following 2/100 Hz EA. This finding is in accordance with the previously suggested notion that the use of EA at alternating low and high frequencies induces a much more potent antinociceptive effect than that induced by a constant frequency stimulation [2, 37]. However, the effect of 2 Hz EA lasted longer than that of 100 or 2/100 Hz EA, as shown elsewhere [2, 8, 38]. 

Lightly anesthetized animals were used in this study to minimize the stress induced by animal restraint, which may have changed the nociceptive threshold [39, 40]. The rats tolerated needle insertion and EA well under 0.5% isoflurane. The TFLs obtained before and during the sham EA conducted under inhalation anesthesia were not significantly different. We conclude that inhalation anesthesia is unlikely to interfere with the EA-induced increase in TFL, as proposed elsewhere [30, 41].

Intrathecal injection of desipramine, fluoxetine, thiorphan, and vigabatrin produced a weak and dose-dependent antinociceptive effect in sham-EA rats. Similarly, weak but not dose-dependent effects were also produced by intrathecal injection of neostigmine and gabapentin.

### 4.2. Changes Produced by Administration of Desipramine, Fluoxetine, Thiorphan, Neostigmine, Gabapentin, or Vigabatrin on the Tail-Flick Test of Sham EA Rats

Desipramine is a selective inhibitor of noradrenaline uptake [42] that has been shown to be antinociceptive in the rat [43] and mouse [44] tail-flick test when used at systemic doses in the range of 5 to 50 *μ*g or when given intrathecally in rats at doses of 6 and 12 *μ*g [45]. However, other tricyclic antidepressants are frequently ineffective against experimental pain [46–48]. Fluoxetine is a selective inhibitor of 5-HT uptake [49, 50], and its effects in animal models of pain are also controversial. Intrathecal fluoxetine produced nonsignificant antinociceptive effects in the mouse tail-flick test [44, 51, 52]. In contrast, intrathecal fluoxetine (5 to 20 *μ*g) produced mild but significant antinociceptive effects in the rat tail-flick test [53]. The different species used in these studies may account for their differences. 

Thiorphan is a synthetic selective inhibitor of endopeptidase 24.11 (enkephalinase) [54], which is an opioid peptide-inactivating enzyme found on the surface of neurons and immune cells [54]. In our study, the effects of thiorphan (16 *μ*g) or neostigmine (0.2 *μ*g) in sham EA rats were very weak but remained above those of the control for at least 30 min. Intrathecal thiorphan (16 *μ*g) did not change the TFL measured 15 min after drug administration in mice [52]. The different species and the time of observation may account for these differences. In contrast, intrathecal injection of neostigmine, an inhibitor of acetylcholinesterase, produces antinociceptive effects in rat models of phasic [55, 56], inflammatory [57], incisional [58], and neuropathic [59] pain and also reduces postoperative pain in human beings [60]. 

Gabapentin was introduced as an anticonvulsant [61], but it has been widely used for the treatment of neuropathic pain [62, 63]. The antinociceptive effect of intrathecal gabapentin has already been demonstrated in the rat tail-flick test [64] and has been confirmed in rat models of neuropathic [65] and postoperative [66, 67] pain.

Vigabatrin is a selective GABA-transaminase inhibitor that slows GABA degradation [25]. The antinociceptive property of systemic administration of vigabatrin (100 to 1200 mg/kg) was shown in mice using the hot plate test [68]; the effect was antagonized by bicuculline and was not prevented by naloxone [69]. 

### 4.3. Changes Induced by Administration of Desipramine, Fluoxetine, Thiorphan, Neostigmine, Gabapentin, or Vigabatrin in the Intensity and Duration of the Effects of EA in the Rat Tail-Flick Test

The maximal possible effect in the tail-flick test was reached after EA in saline-treated rats at all frequencies of stimulation. This finding may explain why none of the administered drugs changed the intensity of the antinociceptive effect of 2, 100, or 2/100 Hz EA. Compared to the antinociceptive effect induced by EA in saline-treated rats, the effect of 2 Hz EA was significantly longer after fluoxetine or neostigmine administration and significantly shorter after gabapentin administration. The effect of 100 Hz EA was also significantly longer after fluoxetine administration, and the effect of 2/100 Hz EA was significantly longer after thiorphan or vigabatrin administration and significantly shorter after gabapentin administration (Table 1).

The intensity and duration of 2 [17] and 2/100 [18] Hz EA-induced antinociception in the rat tail-flick test were both reduced by intrathecal *α*_1_ and mainly *α*_2_-adrenergic antagonists, while 100 Hz EA antinociception resists *α*-adrenergic antagonists [17]. However, the duration of 2, 100, or 2/100 Hz EA-induced antinociception was not changed after desipramine administration. Therefore, it seems that the effects of 2 and 2/100 Hz EA, but not 100 Hz EA, depend on the activation of spinal noradrenergic terminals, and the effects are not changed by increasing the availability of spinal norepinephrine. 

Intrathecal fluoxetine also prolonged 2 and 100 Hz EA antinociception but did not change the duration of 2/100 Hz EA antinociception. In agreement with this result, the spinal content of 5-HT is increased by EA [70, 71], and the blockade of 5-HT synthesis inhibits EA antinociception [3]. Further, the administration of 5-HT antagonists such as cinaserine, cyproheptadine, or methysergide nearly abolished 2 and 100 Hz EA-induced antinociception in mice in the formalin test [10]. Similarly, the antinociception induced by 2 and 100 Hz EA is shortened [17], and 2/100 Hz EA antinociception is blocked [18] by intrathecal methysergide in the rat tail-flick test. These results indicate that spinal serotonergic modulation is involved in the production of antinociception by 2, 100, and 2/100 Hz EA, but only the effects of 2 and 100 Hz EA are changed when serotonin availability in the spinal cord is increased.

Intrathecal thiorphan increased the duration of EA antinociception at all frequencies used in this study. This result agrees with a previous finding that antinociception induced by 2, 100 [17], and 2/100 [18] Hz EA is very sensitive to the intrathecal administration of naloxone. In addition, this result confirms that modulation by spinal opioids is necessary for the production of antinociception by 2, 100, or 2/100 Hz EA.

In neostigmine-treated rats, 2 Hz EA antinociception lasted longer, while 100 and 2/100 Hz EA antinociception was not changed. This result agrees with a previous finding that 2 Hz EA potentiates the antiallodynic effect of intrathecal neostigmine in a rat model of neuropathic pain [59]. Spinal muscarinic cholinergic mechanisms mediating the antinociceptive effect of 2 Hz EA were also demonstrated in a model of neuropathic pain in rats [72]. However, the antinociceptive effect of EA at 2, 100, [17], and 2/100 [18] Hz was weaker and shorter after intrathecal injection of atropine. Therefore, these results indicate that spinal cholinergic modulation is necessary for the production of antinociception by EA at all frequencies used here; however, only the effect of 2 Hz EA was changed by an increase in the availability of spinal acetylcholine. 

Gabapentin reduced the duration of the antinociceptive effects of 2 and 2/100 Hz EA but did not modify the effect of 100 Hz EA. Gabapentin increases the release of GABA from glia and neurons by reversing the GABA transporter [24, 73]. Gabapentin also binds with high affinity to the *α*_2_ *δ* auxiliary subunit of voltage-gated calcium channels [73–75], which seems to be the mechanism through which gabapentin ameliorates neuropathic pain [76]. Most studies have shown that gabapentin reduces the release of various neurotransmitters from synapses in several neuronal tissues [76].

Intrathecal vigabatrin increased the duration of 2/100 Hz EA antinociception. Therefore, an increase in GABA availability at GABAergic synaptic clefts is unlikely to be a mechanism for the changes in EA antinociception observed in rats treated with gabapentin. After bicuculline or phaclofen administration, antinociception induced by 2, 100 [17], and 2/100 [18] Hz EA had a shorter duration and/or was less intense. Together, these results indicate that EA depends on spinal GABAergic modulation; however, only 2/100 Hz EA antinociception is sensitive to an increase in GABA availability in the spinal cord.

## 5. Conclusion

The results presented herein confirm the notion that spinal mechanisms activated during EA antinociception depend on the frequency of stimulation [77, 78]. Antinociception induced by 2 Hz EA lasted longer following intrathecal injection of drugs that increase the spinal availability of norepinephrine, acetylcholine, or GABA. Antinociception induced by 100 Hz EA lasted longer after intrathecal injection of drugs that increase the spinal availability of norepinephrine. Antinociception induced by 2/100 Hz EA lasted longer after intrathecal injection of drugs that increase the spinal availability of endogenous opioids or GABA.

We conclude that 2/100 Hz EA antinociception is not simply the synergistic effect of stimulating alternately at 2 and 100 Hz because its antinociceptive effect is not changed by increasing the spinal availability of serotonin and lasts longer after the administration of vigabatrin. The combination of EA with drugs that increase the availability of neurotransmitters involved in the modulation of spinal nociceptive inputs results in a synergistic antinociceptive effect in the rat tail-flick test. These findings support the use of such combinations in the management of clinical pain.

## Figures and Tables

**Table 1:** Summary of the changes produced by intrathecal drugs on the intensity (int) and duration (dur) of antinociception induced by electroacupuncture (EA) at different frequencies in the rat tail-flick test.

Drug	Dose	Sham	2-Hz EA		100-Hz EA		2/100-Hz EA	
	*μ*g	EA	int	dur	int	dur	int	dur
Desipramine	6	↑	—	—	—	—	—	—
	12	↑↑	—	—	—	—	—	—

Fluoxetine	6	↑	↓ ns	↑	—	↑	—	—
	12	↑↑	—	↑	—	↑↑	—	—

Thiorphan	16	↑	—	—	—	—	—	—
	32	↑↑	—	—	—	—	—	↑

Neostigmine	0.1	↑	—	↑	—	—	—	—
	0.2	↑	—	—	—	—	—	—

Gabapentin	150	—	—	↓	—	—	Block	↓↓
	300	↑	—	↓↓	—	—	—	—

Vigabatrin	0.3	↑	—	—	—	—	—	↑↑
	3.0	↑↑	—	—	—	—	—	↑

Abbreviations: —: no change; ↓: decrease; and ↑: increase (compared to saline-treated rats).
